# Room Temperature Electroluminescence from Tensile-Strained Si_0.13_Ge_0.87_/Ge Multiple Quantum Wells on a Ge Virtual Substrate

**DOI:** 10.3390/ma9100803

**Published:** 2016-09-27

**Authors:** Guangyang Lin, Ningli Chen, Lu Zhang, Zhiwei Huang, Wei Huang, Jianyuan Wang, Jianfang Xu, Songyan Chen, Cheng Li

**Affiliations:** Department of Physics, OSED, Semiconductor Photonics Research Center, Xiamen University, Xiamen 361005, Fujian, China; linguangyang@stu.xmu.edu.cn (G.L.); chenningli9375@163.com (N.C.) zhanglu.225@163.com (L.Z.); 18959206002@163.com (Z.H.); weihuang@xmu.edu.cn (W.H.); wangjianyuan@xmu.edu.cn (J.W.); jfxu@xmu.edu.cn (J.X.); sychen@xmu.edu.cn (S.C.)

**Keywords:** ultra-high vacuum chemical vapor deposition (UHVCVD), tensile strain, SiGe/Ge multiple quantum wells, electroluminescence

## Abstract

Direct band electroluminescence (EL) from tensile-strained Si_0.13_Ge_0.87_/Ge multiple quantum wells (MQWs) on a Ge virtual substrate (VS) at room temperature is reported herein. Due to the competitive result of quantum confinement Stark effect and bandgap narrowing induced by tensile strain in Ge wells, electroluminescence from Γ1-HH1 transition in 12-nm Ge wells was observed at around 1550 nm. As injection current density increases, additional emission shoulders from Γ2-HH2 transition in Ge wells and Ge VS appeared at around 1300–1400 nm and 1600–1700 nm, respectively. The peak energy of EL shifted to the lower energy side superquadratically with an increase of injection current density as a result of the Joule heating effect. During the elevation of environmental temperature, EL intensity increased due to a reduction of energy between L and Γ valleys of Ge. Empirical fitting of the relationship between the integrated intensity of EL (*L*) and injection current density (*J*) with *L*~*J*^m^ shows that the m factor increased with injection current density, suggesting higher light emitting efficiency of the diode at larger injection current densities, which can be attributed to larger carrier occupations in the Γ valley and the heavy hole (HH) valance band at higher temperatures.

## 1. Introduction

In the past several decades, silicon (Si)-based microelectronic integrated circuits (ICs) have achieved abundant progress based on the scaling down rule following Moore’s law [1]. However, as the channel length of metal-oxide-semiconductor field-effect transistor (MOSFET) has approached ~10-nm technology nodes over the past decade [2], RC delay and thermal consumption of metal interconnects have become serious problems retarding further improvement of the performance of Si-based microelectronic ICs [3]. Silicon-based optoelectronic ICs, which incorporate microelectronics and optical devices [4], have been proposed as an effective way to break the bottleneck [5]. To date, most of the components in Si-based optoelectronic ICs with high performance have been demonstrated [6,7,8], but a Si-based light source remains the biggest challenge for the indirect bandgap property of Si. 

For its quasi-direct band structure and compatibility with Si architecture, germanium (Ge) is considered one of the most promising materials for a Si-based light source. Theoretical [9] and experimental [10] results have shown that the energy difference between the L and Γ valleys of Ge is about 140 meV at room temperature and can be further reduced under tensile strain leading to higher light emitting efficiency. N-type heavy doping is considered an alternative way to improve the light emitting efficiency of Ge [11,12], since more electrons can be injected into the Γ valley for n+ Ge under the same injection level compared to intrinsic Ge. Based on these two approaches, photoluminescence (PL) or electroluminescence (EL) [13,14,15,16,17] and even electrically pumped lasing [18] from Ge-on-Si material have been demonstrated. To further improve the light emitting efficiency of the material, SiGe/Ge multiple quantum wells (MQWs) with a Ge-rich (Ge fraction > 0.85) SiGe barrier, which forms a ‘type I’ heterostructure, have been proposed and demonstrated for the strong quantum confinement Stark effect (QCSE) [19,20,21,22]. However, most of the ‘type I’ SiGe/Ge MQWs were grown on a Ge-rich SiGe buffer by a strain-compensated method, which demands the average Si concentration in SiGe/Ge MQWs to be equal to the SiGe buffer, to achieve thick MQWs, resulting in compressive strain in Ge wells. To counteract the detrimental enlargement of energy difference between L and Γ valleys from the QCSE, tensile-strained Ge wells are preferable. So far, few results have been reported about the EL from tensile-strained SiGe/Ge MQWs [23]. 

In this paper, room temperature EL from six periods of tensile-strained Si_0.13_Ge_0.87_/Ge MQWs on a Ge virtual substrate (VS) is reported. Due to the competition effect of the tensile strain and the QCSE in Ge wells, the luminescence wavelength was hauled back at around 1550 nm for telecommunications. The optical properties of tensile-strained Ge wells under various injection currents and temperatures were studied and are discussed here in detail.

## 2. Experiments

### 2.1. Material Growth

**Figure 1:**
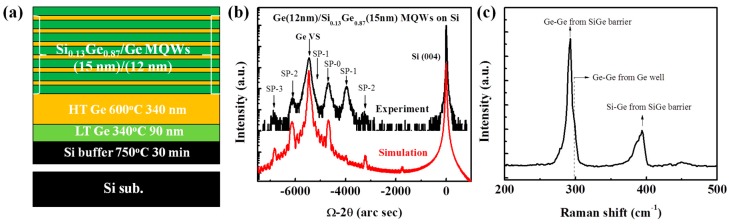
(**a**) Structure diagram for the SiGe/Ge MQWs grown on the Ge-on-Si substrate; (**b**) Corresponding (004) X-ray diffraction rocking curves; and (**c**) Raman spectrum. The strain in Ge wells and Si_0.13_Ge_0.87_ barriers are 0.17% and 0.71%, respectively.

The materials for light emitting diodes were grown on an n-Si (100) substrate with a resistivity of 0.1~1.2 Ω·cm. After wafer cleaning with a modified RCA recipe, the substrate was degassed in a pretreatment chamber at 200 °C for 2 h. Next, the substrate was transferred to a cold-wall ultra-high vacuum chemical vapor deposition (UHVCVD) chamber with a base pressure of 4 × 10^−8^ Pa to remove the native oxide at 850 °C for 30 min. Disilane (Si_2_H_6_) and germane (GeH_4_) were used as a gas source for the deposition of Si and Ge atoms, respectively. A Si buffer layer was firstly grown at 750 °C to bury possible contaminators and imperfect lattices on an initial substrate surface. Next, a 90-nm Ge buffer layer was epitaxially grown at 320 °C to relax the compressive strain resulting from a lattice mismatch and to act as a seed layer. A high-quality Ge VS with a thickness of 340 nm was subsequently grown at 600 °C. After that, six periods of Ge/Si_0.13_Ge_0.87_ multiple quantum wells (MQWs) were grown at 600 °C and were finally capped by a Si_0.13_Ge_0.87_ layer with a thickness of 15 nm. The thicknesses of the Ge wells and SiGe barriers on Ge VS were 12 nm and 15 nm, respectively, and the Ge fraction of SiGe barriers was controlled by adjusting the flux ratio of Si_2_H_6_/GeH_4_. Details of the material growth have been described in previous reports [24,25]. The schematic diagram of the epitaxial structure on the Si substrate, corresponding X-ray diffraction (XRD) rocking curve, and Raman spectrum are exhibited in Figure 1a–c, respectively. Six orders of superlattice satellites can be observed in the XRD curve, indicating high crystal quality and sharp interfaces between Ge and SiGe layers. The strain in the Ge wells and Si_0.13_Ge_0.87_ barriers were extracted and are shown to be 0.17% and 0.71%, respectively.

### 2.2. Device Fabrication

**Figure 2:**
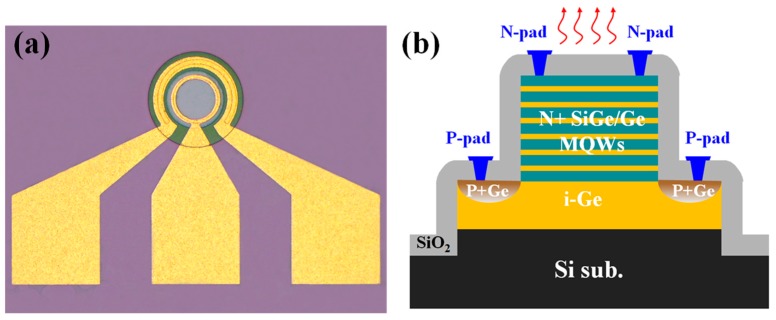
(**a**) Top view image of the fabricated LEDs; (**b**) Corresponding cross section structure.

The material was then fabricated into vertical diodes with double mesas. First, upper mesas with diameters of 100 μm were formed via standard lithography and selective reactive-ion-etch (RIE) of the Si_0.13_Ge_0.87_ cap layer, MQWs, and part of the Ge virtual substrate (VS). The bottom mesas with diameters of 166 μm were fabricated concentrically with the upper mesas via selective RIE of the Ge layer and part of the Si substrate. To obtain p/n junctions, selective phosphorous ion implantation into the upper mesas at 60 keV with a dose of 5 × 10^15^ cm^−2^ and BF_2_^+^ implantation into the bottom mesas at 30 keV with a dose of 5 × 10^15^ cm^−2^ were carried out after the deposition of a 15-nm SiO_2_ layer as a protective layer, respectively. The implanted ions were activated by rapid thermal annealing (RTA) at 650 °C for 30 s in a N_2_ atmosphere. A 300-nm SiO_2_ layer for passivation was then deposited via plasma-enhanced chemical vapor deposition (PECVD), and contact windows were opened by wet etching of the SiO_2_ with buffered-oxide-etchant (BOE). Finally, a 300-nm-thick aluminum (Al) layer was deposited by magnetron sputtering and patterned as electrodes. The top view image of the fabricated LEDs and cross-section structures are exhibited in Figure 2. 

## 3. Results and Discussion

**Figure 3:**
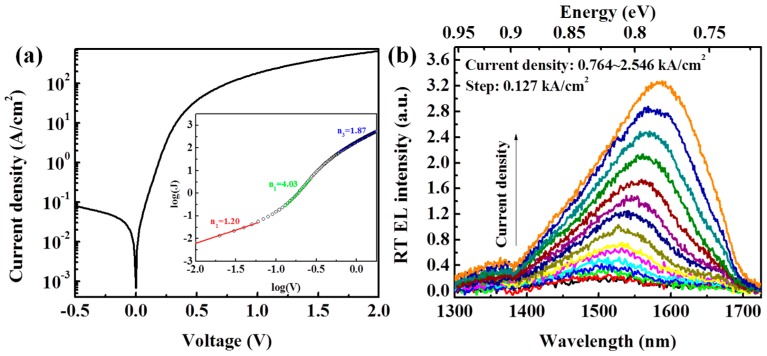
(**a**) Typical *J*–*V* characteristic of the diode; and (**b**) corresponding EL spectra under injection current densities of 0.764–2.546 kA/cm^2^ with a step increase of 0.127 kA/cm^2^ at room temperature.

Figure 3a shows typical *J*–*V* characteristics of the diodes at room temperature. The dependence of current density (*J*) on applied voltage (*V*) can be expressed as [16]

(1) $$J=J_{0}\exp\left[\frac{q\left(V-JAR_{S}\right)}{nk_{B}T}-1\right],$$

where *J*_0_ is the reverse saturation current density, *A* is the junction area, *R*_s_ is the series resistance, *n* is the ideal factor, *k*_B_ is the Boltzmann constant, and *T* is the sample temperature. A good rectify ratio of ~457 at a bias of ±0.5 V was observed. The inset shows the forward *J*–*V* characteristic in a log–log scale marked with extracted ideal factors. At a very small forward bias (*V* < 0.06 V), the ideal factor is about 1.20, indicating the competition mechanism of drift-diffusion and the recombination process [26]. During an increase of forward bias, the recombination current gradually plays a dominating role, leading to an increase in the ideal factor. At a forward bias larger than 0.29 V, the ideal factors exceed 2, reaching 4.03 due to trap-assisted tunneling and carrier leakage in the MQWs [27]. At a larger forward bias (*V* > 1.0 V), the current density is mainly limited by the series resistances, resulting in an ideal factor of 1.87. 

Figure 3b displays the EL spectra from the diode under injection current densities between 0.764 kA/cm^2^ and 2.546 kA/cm^2^ with a step increase of 0.127 kA/cm^2^ at room temperature without any temperature stabilization. The EL was collected by an InGaAs/InP photodetector with a detected limitation of 1725 nm. Luminescence peaks locating at 1500–1600 nm (~0.825 eV) were observed. As injection current density increases, the EL peak intensity becomes stronger, accompanied by an obvious red shift of peak position, suggesting the luminescence mechanism of direct band recombination. 

**Figure 4:**
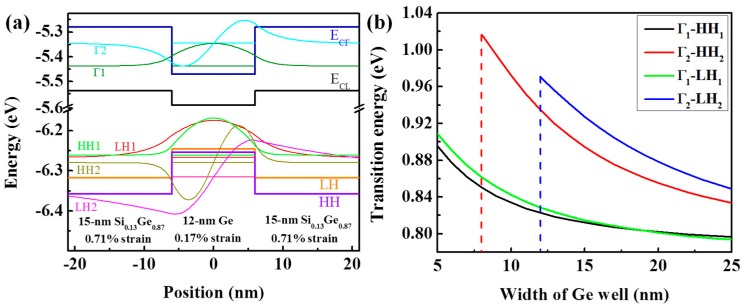
(**a**) Band alignment for a 15-nm Si_0.13_Ge_0.87_/12-nm Ge quantum well and corresponding electronic states; (**b**) Energy variation of Γ1-HH1, Γ2-HH2, Γ1-LH1, and Γ2-LH2 transitions versus the width of a Ge well. The strain in the Ge well and the SiGe barrier used for calculation is 0.17% and 0.71%, respectively.

To confirm the EL mechanisms, electronic states and bandgap structure of the SiGe/Ge MQWs were calculated based on Van de Walle’s deformation theory [28] and quantum physics. The effective mass of heavy holes and electrons in the Γ valley of Si_1-*x*_Ge*_x_* used for calculation are $m_{hh}^{*}=\left(0.53-0.246x\right)m_{0}$ and $m_{\Gamma }^{*}=\left(0.156-0.118x\right)m_{0}$ [19], where *m*_0_ is the rest mass of electron, and *x* is the Ge fraction (0.85–1.00); the direct and indirect bandgaps of Si_1-*x*_Ge*_x_* used for our calculation are 3.4 − 2.60*x* (eV) and 2 − 1.34*x* (eV) [29,30]; other parameters of Si_1-*x*_Ge*_x_* used for calculation are obtained by linear interpolation of the data from Si and Ge as reported previously [28,31,32]. Figure 4a displays the calculated band alignment between a 15-nm Si_0.13_Ge_0.87_ barrier with 0.71% strain and a 12-nm Ge well with 0.17% strain at room temperature. The quantum-confined states (not all) are also exhibited in the figure. As can be seen, the band offsets for the Γ valley, the L valley, the light hole (LH) valance band, and the heavy hole (HH) valance band are 191 meV, 58 meV, 71 meV, and 103 meV, respectively, forming a ‘type I’ band structure. Due to the large band discontinuities at the SiGe/Ge interface, the amount of sublevels in the Γ valley, the L valley, the LH valance band, and the HH valance band are 2, 3, 2, and 4, respectively. Despite the lower energy of the sublevels in the L valley, a considerable amount of electrons are expected to be injected into the sublevels in the Γ valley under a suitable forward bias due to the reduction in the energy difference between the L and Γ valleys (~125 meV), the reduction of the density of the states in the L valley of Ge wells, and the QCSE. Thus, the strong direct band EL from the Ge quantum wells may be observed under large forward biases. 

Figure 4b shows the calculation results for the energy variations of Γ1-HH1, Γ2-HH2, Γ1-LH1, and Γ2-LH2 transitions versus the width of the Ge well. For the Ge well with a width of 12 nm, the transition energies of Γ1-HH1, Γ2-HH2, Γ1-LH1, and Γ2-LH2 are 0.823 eV, 0.935 eV, 0.829 eV, and 0.970 eV, respectively. Thus, the EL peaks at around 0.825 eV come from a direct band transition of Γ1-HH1 and Γ1-LH1. More specifically, the EL peaks mainly come from the Γ1-HH1 transition [33,34], because the luminescence from the Γ1-LH1 transition is transverse-magnetically polarized, and because of the conspicuous preponderance of hole occupation and stronger quantum confinement effect (Figure 4a) in the HH valance band compared with the LH valance band. As injection current density increases, a considerable amount of carriers are injected into the Γ2 and HH2 states of Ge wells. Emission shoulders at 1300–1400 nm from the Γ2-HH2 transition were observed at large injection current densities consequently. In addition, an appreciable part of the injected carriers leaks into the tensile-strained Ge VS under large injection current densities, giving rise to the appearance of emission shoulders at 1600–1700 nm. The EL intensity from MQWs is much stronger than that from the Ge VS due to the following two contributions: (a) the n-type doping in Ge wells, which is a demonstrated benefit of the light emitting of Ge; (b) the QCSE leading to a more concentrated distribution of carriers in the ground state of Ge wells. 

**Figure 5:**
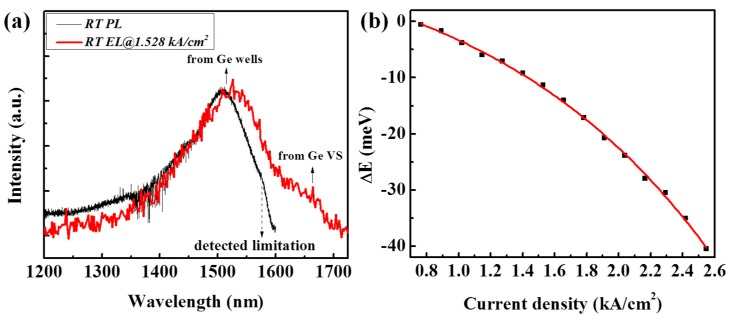
(**a**) Comparison of room temperature photoluminescence (PL) spectrum and EL spectrum under an injection current density of 1.528 kA/cm^2^; (**b**) Reduction of peak energy (ΔE) for EL spectra versus the injection current density (*J*) fitted by $\Delta E\sim J^{2.52}$ .

During the increase of injection current density, the peak positions of EL spectra shift to a lower energy level due to the Joule heating effect. Figure 5a shows the room temperature PL spectrum from the epitaxial Si_0.13_Ge_0.87_/Ge MQWs with a detected limitation of 1575 nm and the EL spectrum under an injection current density of 1.528 kA/cm^2^ for comparison. A slight red shift of the EL peak position is observed. Based on the comparison, the relationship between the red shift of the EL peak positions and injection current densities was extracted and is depicted in Figure 5b. According to Varshni’s law [35], the direct bandgap of Ge shrinks almost linearly with the elevation of the Ge temperature at temperatures above 300 K, which can be expressed as

(2) $$\frac{\Delta E_{g}^{\Gamma }}{\Delta T}\approx -0.462\;\mathrm{meV}/K$$

where $\Delta E_{g}^{\Gamma }$ is the reduction of the Ge direct bandgap, and ΔT is the elevation of the Ge temperature compared to 300 K. Theoretically, Joule heat is proportional to *I*^2^*R*_S_. During the increase of sample temperature, *R*_S_ becomes larger due to the stronger scattering effects of the carriers, such as phonon scattering. Thus, although the heat dissipation rate is larger at higher temperatures for p/n junctions, the temperature of the diode elevates superquadratically with the injection current, resulting in a superquadratic relationship between the energy reduction of the EL peak (ΔE) and injection current density. For our diode, ΔE is proportional to *J*^2.25^. Based on Equation (2), the temperature of the diode elevates to ~87 K under an injection current density of 2.546 kA/cm^2^. 

**Figure 6:**
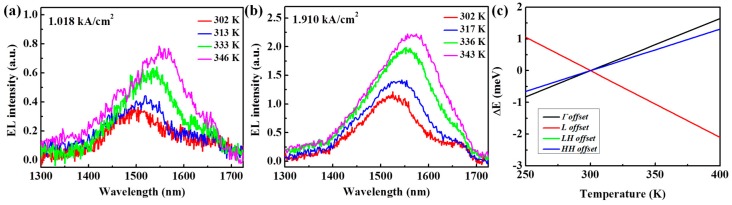
EL spectra measured at different environmental temperatures under constant injection current densities of (**a**) 1.018 kA/cm^2^ and (**b**) 1.910 kA/cm^2^; (**c**) Energy variations of band offsets between the Si_0.13_Ge_0.87_/Ge interface and the environmental temperature (250–400 K). The variations of the band offsets for the LH valance band and the Γ valley are almost identical.

To further study the temperature dependence of optical properties of the diode, EL spectra under constant injection current densities of 1.018 kA/cm^2^ and 1.910 kA/cm^2^ at different environmental temperatures (302–346 K) were measured, as exhibited in Figure 6a,b, respectively. During the increase in the environmental temperature, EL from both the Ge wells and the Ge VS becomes stronger. The energy variation of band offsets between the Si_0.13_Ge_0.87_ barriers and Ge well interface and the temperature (250–400 K) were calculated based on [36] to help analyze the mechanisms, as shown in Figure 6c. The band offset of the L valley slightly decreases with the increase in temperature, while the rest of the band offsets are enlarged slightly with the elevation of temperature. Compared to the band offsets at 300 K, the energy variation of all band offsets is within ~1 meV at 350 K, having a negligible effect on the sublevels in the Ge wells. On the other hand, the energy difference between the L and Γ valleys of Ge shrinks at a rate of ~0.2 meV/K [36], leading to an energy reduction of ~10 meV at 350 K between the L and Γ valleys. Thus, the enhancement of EL intensity for the diode under a constant injection current density at a higher environmental temperature should be mainly attributed to the energy reduction between the L and Γ valleys, which results in a larger occupation of electrons in the Γ valley [20]. 

**Figure 7:**
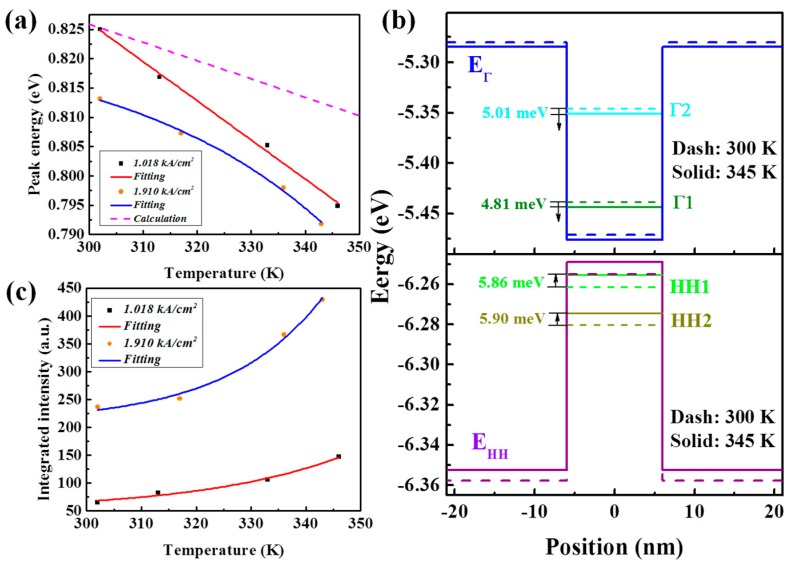
(**a**) Energy variation of EL peaks under constant injection current densities of 1.018 kA/cm^2^ and 1.910 kA/cm^2^ versus the environmental temperature. The energy of the Γ1-HH1 transition from the calculated results is also plotted; (**b**) Band alignment for the MQWs at 345 K from calculated results based on [36]; (**c**) Variation of integrated EL intensity under the constant injection current densities of 1.018 kA/cm^2^ and 1.910 kA/cm^2^ versus the environmental temperature.

Figure 7a shows the energy variation of the EL peaks under the constant injection current densities of 1.018 kA/cm^2^ and 1.910 kA/cm^2^ versus the environmental temperature. Based on [36], the band alignment for the MQWs at 345 K was calculated and is displayed in Figure 7b. Compared to the circumstance at 300 K, the energy of the Γ1 sublevel is reduced by 4.81 meV, while the energy of the HH1 sublevel is elevated by 5.86 meV, leading to a reduction of Γ1-HH1 transition energy by 10.67 meV. Detailed energy variation of the Γ1-HH1 transition versus the environmental temperature from the calculated results, which decreases with the elevation of temperature, is plotted in Figure 7a with a dashed line. As can be seen, the experimental data differs from the calculated results due to the Joule heating effect of the injection current. During the increase in environmental temperature, this difference is enlarged dramatically. In addition, the discrepancy of the EL peak energy under the injection current densities of 1.018 kA/cm^2^ and 1.910 kA/cm^2^ diminishes at a higher temperature. These results suggest that a stronger Joule heating effect at higher temperatures agrees well with the superquadratic dependence of the energy reduction of the direct bandgap of Ge on injection current density, as mentioned above. Since the Joule heating effect is stronger for the diode under a larger injection current density, the enhancement of the integrated EL intensity for the diode under an injection current density of 1.910 kA/cm^2^ is greater than that under an injection current density of 1.018 kA/cm^2^ at elevated environmental temperatures, as shown in Figure 7b.

**Figure 8:**
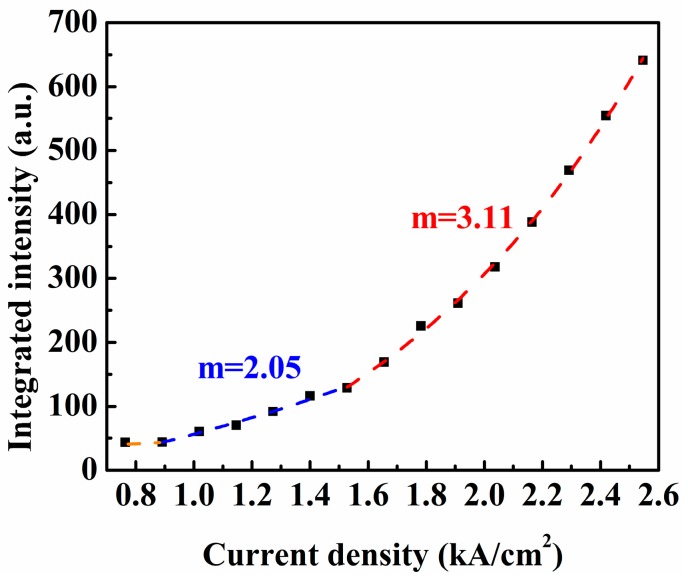
The relationship between the integrated intensity (*L*) from diode and injection current density (*J*) characterized empirically by *L*~*J*^m^.

Figure 8 shows the relationship between the integrated EL intensity (L) and injection current density (J) of the diodes at room temperature characterized empirically by *L*~*J*^m^ in a log–log scale. The extracted m factors are also marked in the figure. Marked luminescence was observed for *J* ≥ 0.891 kA/cm^2^. During the increase of injection current density, the m factor increases from 2.05 for *J* < 0.14 kA/cm^2^ to 3.11 for *J* > 0.14 kA/cm^2^, suggesting the higher efficiency of EL from the diode at a larger injection current density. The improvement of luminescence efficiency was due to the temperature elevation of the diode as a result of the Joule heating effect, which results in more carriers injected into the Γ valley and the HH valance band as mentioned above. 

## 4. Conclusions 

In summary, direct band electroluminescence (EL) from tensile-strained Si_0.13_Ge_0.87_/Ge multiple quantum wells (MQWs) at room temperature was reported herein. The MQWs were grown on a 0.07% tensile-strained Ge virtual substrate via a UHVCVD system. Electroluminescence from Γ1-HH1 transition in 12-nm-thick Ge wells was observed at around 1550 nm, as a competitive result of the quantum confinement Stark effect and the bandgap narrowing induced by tensile strain in Ge wells. As the injection current density increased, additional emission shoulders from Γ2-HH2 transition in Ge wells and Ge VS appeared at around 1300–1400 nm and 1600–1700 nm, respectively. The peak energy of EL decreased superquadratically with the increase in injection current density ($\Delta E\sim J^{2.52}$) due to the Joule heating effect of the injection current. At temperatures between 300 K and 350 K, the energy variation of band offsets between Si_0.13_Ge_0.87_ barriers and Ge wells can be neglected, while the energy difference between L and Γ valleys shrinks significantly during the elevation of temperature, resulting in the stronger EL intensity of the diode under constant injection current densities accompanied with a red shift of the peak position. Empirical fitting of the relationship between the integrated intensity (*L*) of EL and injection current density (*J*) with *L*~*J*^m^ shows that the m factor increases with injection current density, suggesting higher light emitting efficiency of the diode at larger injection current densities, which can be attributed to larger carrier occupations in the Γ valley and the HH valance band at higher temperature.

## References

[1] Moore G.E. (1965). Cramming more components onto integrated circuits. Electronics.

[2] Ishikawa Y., Saito S. (2014). Ge-on-Si photonic devices for photonic-electronic integration on a Si platform. IEICE Electron. Express.

[3] Haensch W., Nowak E.J., Dennard R.H., Solomon P.M., Bryant A., Dokumaci O.H., Kumar A., Wang X., Johnson J.B., Fischetti M.V. (2006). Silicon CMOS devices beyond scaling. IBM J. Res. Dev..

[4] Kirchain R., Kimerling L. (2007). A roadmap for nanophotonics. Nat. Photonics.

[5] Lipson M. (2004). Overcoming the limitations of microelectronics using Si nanophotonics: Solving the coupling, modulation and switching challenges. Nanotechnology.

[6] Maeda T., Ikeda K., Nakaharai S., Tezuka T., Sugiyama N., Moriyama Y., Takagi S. (2005). High mobility Ge-on-insulator p-channel MOSFETs using Pt germanide Schottky source/drain. IEEE Electron Device Lett..

[7] Kang Y., Liu H.-D., Morse M., Paniccia M.J., Zadka M., Litski S., Sarid G., Pauchard A., Kuo Y.-H., Chen H.-W. (2009). Monolithic germanium/silicon avalanche photodiodes with 340 GHz gain–bandwidth product. Nat. Photonics.

[8] Kuo Y.-H., Lee Y.K., Ge Y.S., Ren S., Roth J.E., Kamins T.I., Miller D.A.B., Harris J.S. (2006). Quantum-confined Stark effect in Ge/SiGe quantum wells on Si for optical modulators. IEEE J. Sel. Top. Quantum Electron..

[9] Sun X.C., Liu J.F., Kimerling L.C., Michel J. (2010). Toward a germanium laser for integrated silicon photonics. IEEE J. Sel. Top. Quantum Electron..

[10] Ghrib A., de Kersauson M., EI Kurdi M., Jakomin R., Beaudoin G., Sauvage S., Fishman G., Ndong G., Chaigneau M., Ossikovski R. (2012). Control of tensile strain in germanium waveguides through silicon nitride layers. Appl. Phys. Lett..

[11] Cai Y., Han Z.H., Wang X.X., Camacho-Aguilera R.E., Kimerling L.C., Michel J., Liu J.F. (2013). Analysis of threshold current behavior for bulk and quantum-well germanium laser structures. IEEE J. Sel. Top. Quantum Electron..

[12] Lin G.Y., Wang C., Li C., Chen C.W., Huang Z.W., Huang W., Chen S.Y., Lai H.K., Jin C.Y., Sun J.M. (2016). Strong electroluminescence from direct band and defects in Ge n+/p shallow junctions at room temperature. Appl. Phys. Lett..

[13] Nam D., Sukhdeo D., Cheng S.-L., Roy A., Huang K.C.-Y., Brongersma M., Nishi Y., Saraswat K. (2012). Electroluminescence from strained germanium membranes and implications for an efficient Si-compatible laser. Appl. Phys. Lett..

[14] Oehme M., Gollhofer M., Widmann D., Schmid M., Kaschel M., Kasper E., Schulze J. (2013). Direct bandgap narrowing in Ge LED’s on Si substrates. Opt. Express.

[15] Sun X.C., Liu J.F., Kimerling L.C., Michel J. (2009). Room-temperature direct bandgap electroluminesence from Ge-on-Si light-emitting diodes. Opt. Lett..

[16] Hu W.X., Cheng B.W., Xue C.L., Xue H.Y., Su S.J., Bai A.Q., Luo L.P., Yu Y.D., Wang Q.M. (2009). Electroluminescence from Ge on Si substrate at room temperature. Appl. Phys. Lett..

[17] Huang S.H., Lu W.F., Li C., Huang W., Lai H.K., Chen S.Y. (2013). A CMOS-compatible approach to fabricate an ultra-thin germanium-on-insulator with large tensile strain for Si-based light emission. Opt. Express.

[18] Camacho-Aguilera R.E., Cai Y., Patel N., Bessette J.T., Romagnoli M., Kiemerling L.C., Michel J. (2012). An electrically pumped germanium laser. Opt. Express.

[19] Kuo Y.-H., Lee Y.K., Ge Y.S., Ren S., Roth J.E., Kamins T., Miller D.A.B., Harris J.S. (2005). Strong quantum-confined Stark effect in germanium quantum-well structures on silicon. Nature.

[20] Wu P.H., Dumcenco D., Huang Y.S., Hsu H.P., Lai C.H., Lin T.Y., Chrastina D., Isella G., Gatti E., Tiong K.K. (2012). Above-room-temperature photoluminescence from a strain-compensated Ge/Si_0.15_Ge_0.85_ multiple-quantum-well structure. Appl. Phys. Lett..

[21] Fei E.T., Chen X.C., Zang K., Huo Y.J., Shambat G., Miller G., Liu X., Dutt R., Kamins T.I., Vuckovic J. (2015). Investigation of germanium quantum-well light sources. Opt. Express.

[22] Liu Z., Hu W.X., Li C., Li Y.M., Xue C.L., Li C.B., Zuo Y.H., Cheng B.W., Wang Q.M. (2012). Room temperature direct-bandgap electroluminescence from n-type strain-compensated Ge/SiGe multiple quantum wells. Appl. Phys. Lett..

[23] He C., Liu Z., Zhang X., Huang W.Q., Xue C.L., Cheng B.W. (2014). Direct-bandgap electroluminescence from tensile-strained Ge/SiGe multiple quantum wells at room temperature. Chin. Phys. B.

[24] Zhou Z.W., Li C., Lai H.K., Chen S.Y., Yu J.Z. (2008). The influence of low-temperature Ge seed layer on growth of high-quality Ge epilayer on Si(100) by ultrahigh vacuum chemical vapor deposition. J. Cryst. Growth.

[25] Chen Y.H., Li C., Zhou Z.W., Lai H.K., Chen S.Y., Ding W.C., Cheng B.W., Yu Y.D. (2009). Room temperature photoluminescence of tensile-strained Ge/Si_0.13_Ge_0.87_ quantum wells grown on silicon-based germanium virtual substrate. Appl. Phys. Lett..

[26] Sah C.T., Noyce R.N., Shockley W. (1957). Carrier generation and recombination in pn junctions and pn junction characteristics. Proc. IRE.

[27] Mayes K., Yasan A., McClintock R., Shiell D., Darvish S.R., Kung P., Razeghi M. (2004). High-power 280 nm AlGaN light-emitting diodes based on an asymmetric single-quantum well. Appl. Phys. Lett..

[28] Van de Walle C.G. (1989). Band lineups and deformation potentials in the model-solid theory. Phys. Rev. B.

[29] Bassani F., Brust D. (1963). Effect of alloying and pressure on the band structure of germanium and silicon. Phys. Rev..

[30] Weber J., Alonso M.I. (1989). Near-band-gap photoluminescence of Si-Ge alloys. Phys. Rev. B.

[31] Ishikawa Y., Wada K., Liu J.F., Cannon D.D., Luan H.C., Michel J., Kimerling L.C. (2005). Strain-induced enhancement of near-infrared absorption in Ge epitaxial layers grown on Si substrate. J. Appl. Phys..

[32] Wortman J.J., Evans R.A. (1965). Young’s modulus, shear modulus, and poisson’s ratio in silicon and germanium. J. Appl. Phys..

[33] Chaisakul P., Marris-Morini D., Isella G., Chrastina D., Izard N., Roux X.L., Edmond S., Coudevylle J.-R., Vivien L. (2011). Room temperature direct gap electroluminescence from Ge/Si_0.15_Ge_0.85_ multiple quantum well waveguide. Appl. Phys. Lett..

[34] Sun X.C., Liu J.F., Kimerling L.C., Michel J. (2009). Direct gap photoluminescence of n-type tensile-strained Ge-on-Si. Appl. Phys. Lett..

[35] Varshni Y.P. (1967). Temperature dependence of the energy gap in semiconductors. Physica.

[36] Lautenschlager P., Allen P.B., Cardona M. (1985). Temperature dependence of band gaps in Si and Ge. Phy. Rev. B.

